# Changes of Photosynthetic Parameters in Melatonin-Treated Wheat Subjected to Drought

**DOI:** 10.3390/plants13233414

**Published:** 2024-12-05

**Authors:** Dessislava Todorova, Svetoslav Anev, Martin Iliev, Margarita Petrakova, Iskren Sergiev

**Affiliations:** 1 Institute of Plant Physiology and Genetics, Bulgarian Academy of Sciences, Acad. Georgi Bonchev Street, Bldg. 21, 1113 Sofia, Bulgaria; dessita@bio21.bas.bg (D.T.); martin.vl.iliev@gmail.com (M.I.); margarita.p02@abv.bg (M.P.); 2 Department Dendrology, Faculty of Forestry, University of Forestry, 10 Sveti Kliment Ohridski Blvd, 1756 Sofia, Bulgaria; svetoslav.anev@ltu.bg

**Keywords:** *Triticum aestivum*, drought, melatonin, chlorophyll *a* fluorescence, photosynthesis rate

## Abstract

Drought stress affects many aspects of plant biochemistry, with photosynthesis being one the most significantly impaired physiological processes. Melatonin is a natural antioxidant with growth-regulating properties in plants. Its diverse physiological functions have been extensively studied in recent decades. Changes in leaf gas exchange and chlorophyll *a* fluorescence parameters were investigated in young wheat plants (*Triticum aestivum* L.) cv. Fermer and cv. Gines which were characterized to differ in their responses to drought, with cv. Gines being more tolerant than cv. Fermer. The plants were subjected to drought for five days by withholding their water supply. Melatonin was applied as a root supplement to the irrigation water before or after the drought period. Analyses were performed before and at the end of the stress period, as well as during the recovery phase. Changes in leaf pigment content, photosynthetic rate, stomatal conductance, and transpiration, as well as some chlorophyll *a* fluorescence parameters, were recorded. Melatonin alone did not cause considerable changes in the measured traits. We found a significant decrease in leaf gas exchange parameters, F_v_/F_m_ and F_v_/F_0_ values, and leaf pigments due to drought, especially in cv. Fermer. The data show that the application of melatonin favorably influenced the efficiency of the photosynthetic apparatus under water deprivation and during plant recovery. The pre-treatment with melatonin maintained the photosynthesis-related parameters closer to the control levels during the stress period. Both melatonin treatments supported the recovery of photosynthesis when the water supply was restored and the post-drought treatment showed a similar but weaker effect than pre-drought treatment.

## 1. Introduction

Throughout their lifetime, crops are subjected to various unfavorable environmental conditions that result in significant reductions in vegetative growth and yield. As a consequence of global climate change, water scarcity is becoming a serious problem in many regions, including areas traditionally known for regular rainfall. Water deficit often poses challenges in farming areas and causes significant biochemical, physiological, and morphological changes in plants [1,2,3,4]. One of the outcomes is the buildup of reactive oxygen species (ROS), which can harm large biomolecules and biological membranes in a cascade manner [5]. Further, soil aridity results in a lack of water in plant cells that provokes a reduction of turgor pressure and closing of the stomata to prevent additional water loss. As a result, limitations of transpiration and notable reductions in the rate of photosynthesis occur. Under conditions of limited water availability, the flow of electrons through PS II is restricted [6]. Numerous investigations have indicated that drought stress adversely affected the oxygen-releasing component of PSII, and disrupted the association of light-harvesting complexes with photosynthetic reaction centers of PSII [7,8,9,10].

Along with rice and corn, wheat is an essential fundamental crop for the human population [11]. Although it can withstand certain drought conditions, extended periods of water deficit can cause severe issues and adverse alterations in plants, leading to a downturn in productivity [12]. In the perspective of increasing climatic unpredictability, there is a growing interest in developing strategies to minimize the negative impact of drought. An innovative approach is the application of natural, ecologically safe biostimulators. Melatonin (N-acetyl-5-methoxytryptamine) is a ubiquitous metabolite with pleiotropic regulatory roles in plant biochemistry. Recently, melatonin has been widely researched as a plant growth regulator or biostimulator [5,13,14,15,16,17]. Additionally, melatonin is known as a successful natural ROS remover and antioxidant defense booster in various stress situations such as extreme temperatures, high salt and toxic metals levels, exposure to UV, water deficit, etc. [13,15,18,19,20]. It is documented that after the exogenous application of melatonin, the efficiency of photosynthesis is significantly preserved under drought conditions by controlling stomata closure and transpiration, and also by preserving the functionality of chloroplast grana by reducing lipid peroxidation and retarding the degradation of photosynthetic pigments [21,22,23,24,25]. In their study Li et al. [26] demonstrated that root-applied melatonin influenced the photosynthesis of two wheat cultivars with contrasting drought tolerance in a different manner. In our recent study [27], we found that the biochemical responses of wheat cultivars Fermer and Gines to melatonin treatment under drought and during recovery are associated to some extent with the cultivar’s tolerance to drought. Cv. Gines was previously reported to be more drought tolerant than cv. Fermer [28,29]. Based on these findings we were interested to investigate if there are differences in the effects of melatonin on photosynthesis performance in these cultivars under drought and during recovery. 

In the majority of publications, the application of melatonin is documented as a preventive measure applied prior to drought [19,22,30]. Our literature survey revealed little available evidence regarding the effects of melatonin applied to wheat as a plant recovery aid following water deficit [27]. The current study aimed to compare the effects of exogenous melatonin applied prior to or after drought stress on photosynthesis-related parameters in two Bulgarian varieties of wheat. Leaf pigment content, leaf gas exchange rate, and chlorophyll *a* fluorescence indices were measured in drought-treated and melatonin-supplied plants at the end of stress, as well as after the recovery period. Our results broaden the knowledge regarding the potential of melatonin to facilitate plant recovery after drought.

## 2. Results

### 2.1. Effects of Drought and Melatonin on Wheat Leaf Pigment Content

**Figure 1:**
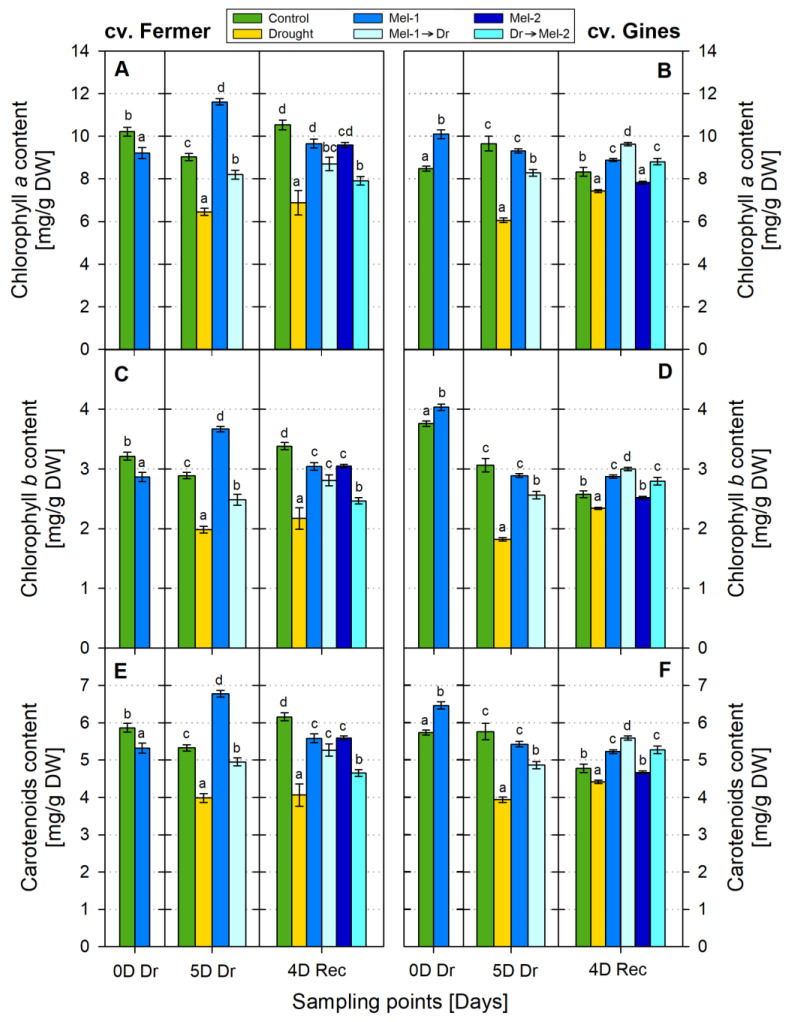
Leaf pigment content in wheat plants: chlorophyll *a* (**A**,**B**), chlorophyll *b* (**C**,**D**), and carotenoids (**E**,**F**) in cv. Fermer (**A**,**C**,**E**) and cv. Gines (**B**,**D**,**F**) at the beginning of drought (0D Dr), at the fifth day of drought (5D Dr), and at the fourth day of recovery (4D Rec). The abbreviations within the legend denote the following: Mel-1—melatonin control before drought, Mel-2—melatonin control after drought, Mel-1 → Dr—melatonin pre-application before drought, Dr → Mel-2—melatonin post-application after drought. Different small letters within each panel section indicate significant differences between the treatments. Error bars designate ±SE.

Phenotype alterations due to drought correlated well with the changes in the amount of leaf pigment. Drought stress significantly decreased leaf pigment content in both wheat cultivars (Figure 1). The decrease detected was by 28% for chlorophyll *a* (Figure 1A), by 31% for chlorophyll *b* (Figure 1C), and by 25% for carotenoids (Figure 1E) in cv. Fermer. In cv. Gines after five days of drought the leaf pigments were decreased by 37% (Figure 1B), by 40% (Figure 1D), and by 32% (Figure 1F).

After water supply restoration, all leaf pigment tended to increase, and at day four of recovery, the content almost reached control levels in cv. Gines. However, in cv. Fermer the levels of leaf pigment in drought-treated wheat were still lower than those in the control plants. Although melatonin applied alone caused some fluctuations in the amount of leaf pigment, its supply prior to or after drought stress was capable of retarding leaf pigment losses. The level of leaf pigment in combine-treated plants (either Mel + Dr or Dr + Mel) was significantly higher when compared with that of the drought-treated plants after five days of stress and at the end of the recovery period.

### 2.2. Effects of Drought and Melatonin on Wheat Leaf Gas Exchange Parameters 

**Figure 2:**
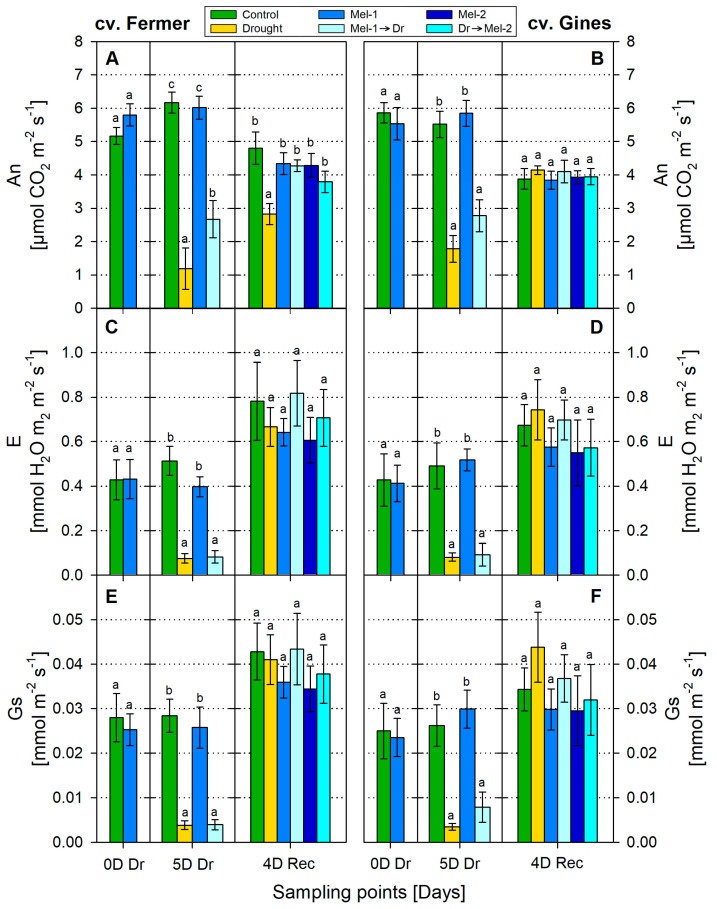
Leaf gas exchange parameters in wheat: net photosynthesis rate An, μmol CO_2_ m^−2^ s^−1^ (**A**,**B**), transpiration rate E, mmol H_2_O m^−2^ s^−1^ (**C**,**D**), and stomatal conductance Gs, mmol m^−2^ s^−1^ (**E**,**F**) in cv. Fermer (**A**,**C**,**E**) and cv. Gines (**B**,**D**,**F**) at the beginning of drought (0D Dr), at the fifth day of drought (5D Dr), and at the fourth day of recovery (4D Rec). The abbreviations within the legend denote the following: Mel-1—melatonin control before drought, Mel-2—melatonin control after drought, Mel-1 → Dr—melatonin pre-application before drought, Dr → Mel-2—melatonin post-application after drought. Different small letters within each panel section indicate significant differences between the treatments. Error bars designate ±SE.

Under normal irrigation, melatonin did not cause substantial alteration in leaf gas exchange parameters studied in both wheat cultivars (Figure 2).

The five-day drought stress period significantly declined net photosynthesis (An), transpiration rate (E), and stomatal conductance (Gs) in both cv. Fermer and cv. Gines plants. The decrease detected of An was 81% in cv. Fermer (Figure 2A) and 71% in cv. Gines (Figure 2B). Accordingly, the transpiration rate was inhibited by 85% and by 84% in cv. Fermer (Figure 2C) and in cv. Gines (Figure 2D), respectively, while Gs was diminished by 87% in both cultivars (Figure 2E,F). After transferring plants to a normal water supply, leaf gas exchange parameters showed a tendency to increase. At the fourth day of recovery, only An in cv. Fermer was still 40% lower than the control photosynthesis rate. Melatonin application prior to or after drought stress aided better recovery of the net photosynthesis rate, especially in cv. Fermer.

### 2.3. Effects of Drought and Melatonin on Wheat Chlorophyll a Fluorescence Parameters

**Figure 3:**
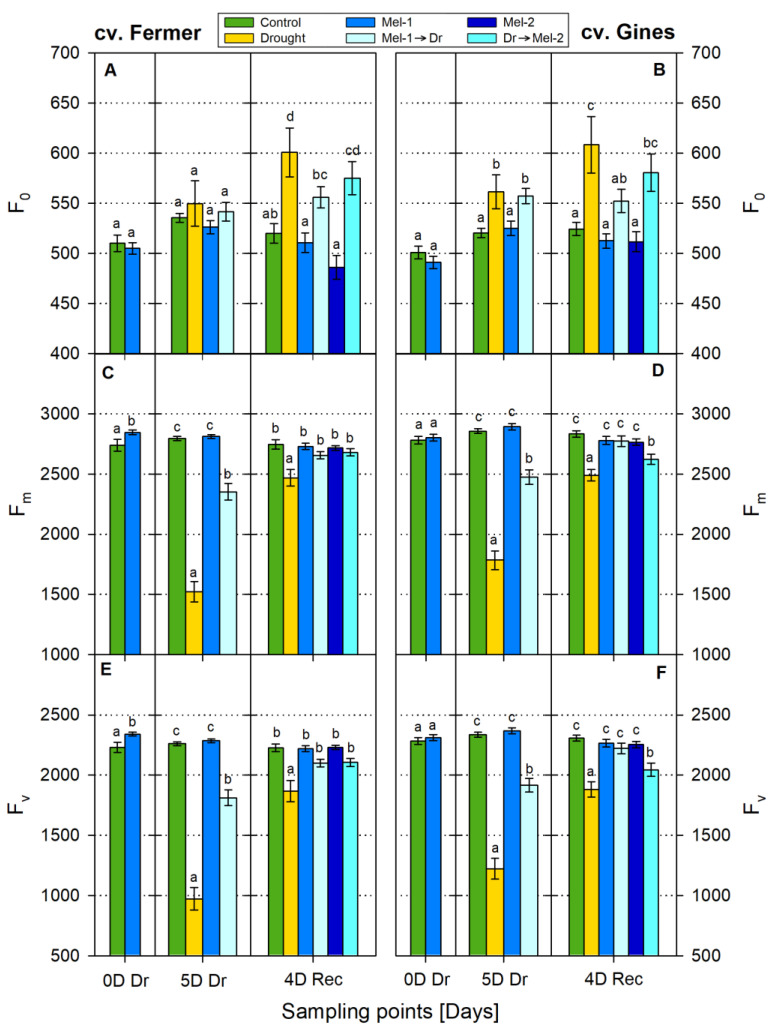
Primary chlorophyll a fluorescence parameters in wheat plants: minimal fluorescence F0 (**A**,**B**), maximal fluorescence Fm (**C**,**D**), and variable fluorescence Fv (**E**,**F**) in cv. Fermer (**A**,**C**,**E**) and cv. Gines (**B**,**D**,**F**) at the beginning of drought (0D Dr), at the fifth day of drought (5D Dr), and at the fourth day of recovery (4D Rec). The abbreviations within the legend denote the following: Mel-1—melatonin control before drought, Mel-2—melatonin control after drought, Mel-1 → Dr—melatonin pre-application before drought, Dr → Mel-2—melatonin post-application after drought. Different small letters within each panel section indicate significant differences between the treatments. Error bars designate ±SE.

**Figure 4:**
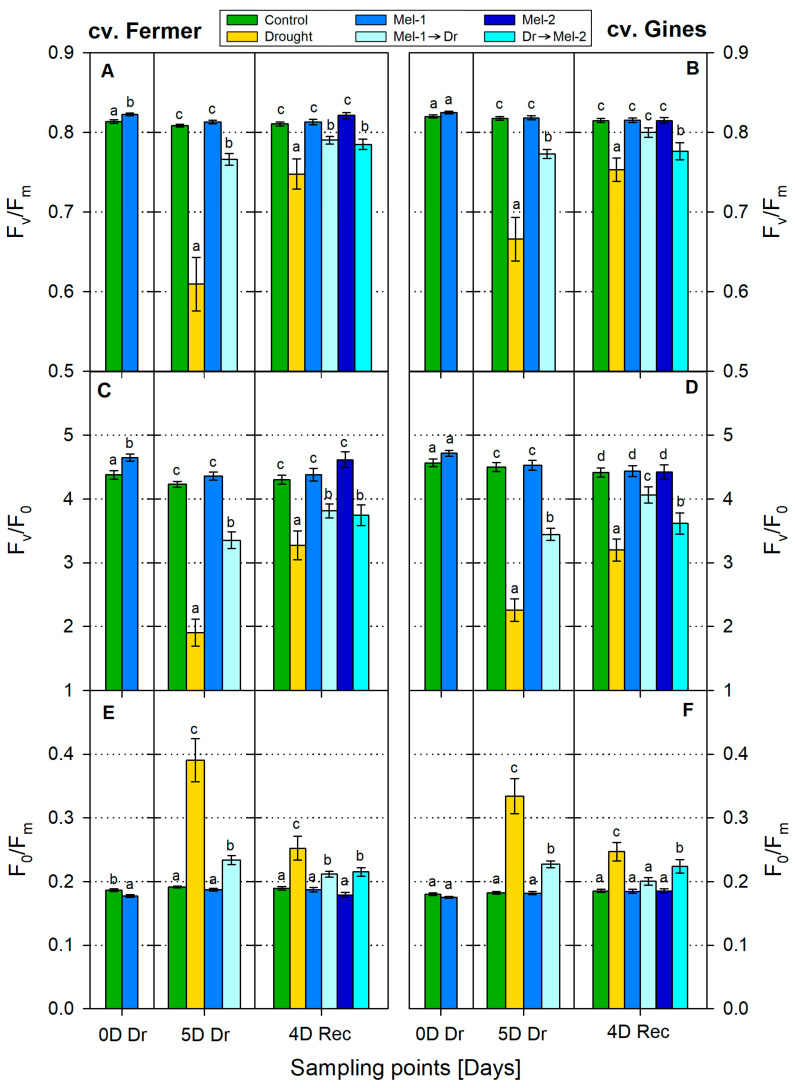
Derived chlorophyll *a* fluorescence parameters in wheat plants: maximum quantum yield of photosystem II F_v_/F_m_ (**A**,**B**), electron transport outside Q_A_^−^ F_v_/F_0_ (**C**,**D**), and quantum efficiency of energy dissipation as heat F_0_/F_m_ (**E**,**F**) in cv. Fermer (**A**,**C**,**E**) and cv. Gines (**B**,**D**,**F**) at the beginning of drought (0D Dr), at the fifth day of drought (5D Dr), and at the fourth day of recovery (4D Rec). The abbreviations within the legend denote the following: Mel-1—melatonin control before drought, Mel-2—melatonin control after drought, Mel-1 → Dr—melatonin pre-application before drought, Dr → Mel-2—melatonin post-application after drought. Different small letters within each panel section indicate significant differences between the treatments. Error bars designate ±SE.

Melatonin applied alone did not cause a substantial alteration in the primary (Figure 3) or derived (Figure 4) parameters of chlorophyll *a* fluorescence. Drought stress provoked a minor increase in F_0_ in parallel with a substantial decrease in F_m_ (by 46% and 38%) and F_v_ (by 57% and 48%) in cv. Fermer and cv. Gines wheat, respectively (Figure 3). During the recovery period, these primary parameters tended to increase in drought-treated seedlings, including F_0_. After four days of recovery, F_m_ and F_v_ of the drought-treated plants were still below the control levels of cv. Fermer and cv. Gines by 10–12% and 16–20%, respectively. F_0_ was higher by 16% in both cv. Fermer and cv. Gines as compared with the respective control plants.

Regarding the derived parameters of chlorophyll *a* fluorescence, a substantial decrease was detected in cv. Fermer and cv. Gines after five days of drought. F_v_/F_m_ was decreased by 25% and 19% (Figure 4A,B), and F_v_/F_0_—by 55% and 50% (Figure 4C,D), respectively. This was accompanied by an increase in F_0_/F_m_ (Figure 4E,F) by 104% and 83% in cv. Fermer and in cv. Gines, respectively. After restoring normal irrigation, the chlorophyll *a* fluorescence parameters tended to recover to physiological levels. At 4D of recovery, F_v_/F_m_ was only 8% below the control (in both cultivars), F_v_/F_0_ was still reduced by 24% and 28% (in cv. Fermer and cv. Gines, respectively), while F_0_/F_m_ was higher by 33% in both drought-treated cultivars.

Melatonin applied prior to or after the drought stress period was capable of diminishing the negative consequences of drought stress in relation to basic and derived chlorophyll *a* fluorescence indices. This was obvious not only during the stress period (melatonin pre-treatment) but also during the recovery period when post-treatment with melatonin accelerated the recovery of chlorophyll *a* fluorescence parameters, although this effect was less significant than that of the melatonin pre-treatment. 

Spider plot presentations of the studied photosynthesis-related parameters normalized to the control are presented in Supplementary Figures S1 and S2. 

### 2.4. Effects of Drought and Melatonin on Wheat Phenotype 

**Figure 5:**
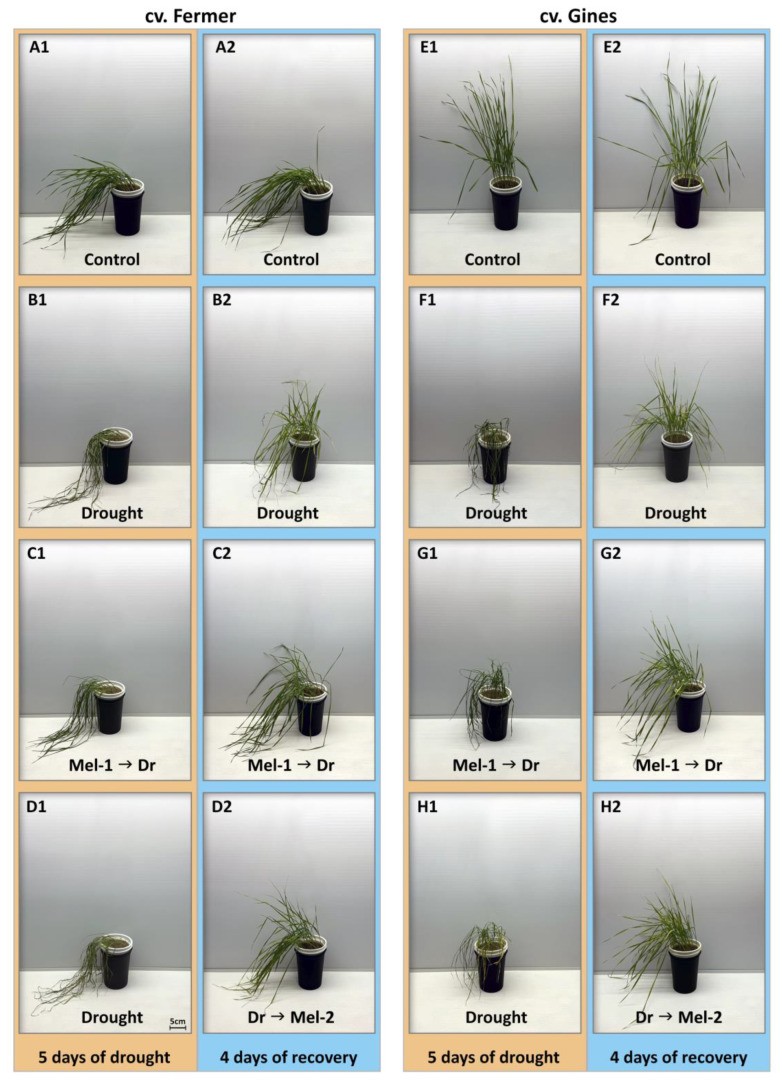
Phenotype changes in wheat plants cv. Fermer (panels (**A**–**D**)) and cv. Gines (panels (**E**–**H**)). Photos labeled with identical letters represent the same pot photographed at the fifth day of drought (number (**1**)), and at the fourth day of recovery (number (**2**)). Treatments: control (panels (**A**,**E**)), drought (panels (**B**,**F**)), melatonin prior to drought (Mel-1 → Dr; panels (**C**,**G**)), and melatonin after drought (Dr → Mel-2; panels (**D**,**H**)).

Figure 5 presents photography of the following treatments: control, drought-treated, melatonin pretreated and subjected to drought, and drought-treated and supplemented with melatonin after drought. Images were taken at the end of stress and after four days of recovery. Drought stress imposed for five days caused typical changes in the phenotype, and obvious wilting and yellowing of the leaves of both cultivars were observed (Figure 5(B1,F1)), which was more pronounced in cv. Fermer. Melatonin applied before drought prevented these alterations to some degree in combine-treated seedlings (Figure 5(C1,G1)). After restoration of their water supply, the leaves of drought-stressed plants regained their turgid state after four days of recovery (Figure 5(B2,F2)), except for some leaf segments which were irreversibly damaged, especially in cv. Fermer. The plants treated with melatonin applied before (Figure 5(C2,G2)) or after drought stress (Figure 5(D2,H2)) performed better when compared with the respective drought-treated individuals (Figure 5(B2,F2)). Melatonin applied alone did not provoke alterations in the phenotype of individuals of both wheat cultivars. A similar observation was reported in our previous research [31].

## 3. Discussion

Photosynthesis is a complex multifaceted physiological process, but nonetheless its inherent flexibility exhibits a significant degree of vulnerability to fluctuations in various environmental conditions, one of the most critical of which is water deficit, a factor that can drastically affect the overall efficiency of this essential process. The application of different types of phytohormones and plant growth regulators may alleviate the negative effects of drought stress on photosynthesis-related components in various crops [32]. In this study, we attempted to modulate the negative effects of water deficit on photosynthesis by using melatonin as root supplement before drought as a preventive measure to withstand stress. Additionally, we applied melatonin during the rehydration period, which helped to accelerate the recovery of photosynthesis in drought-stressed plants. 

It is well documented that drought hinders the growth and development of plants [33,34]. Although wheat is a relatively drought-tolerant crop, we detected a worsening of the morphological traits of the seedlings grown under drought stress conditions (Figure 5) which is similar to the reports of other researchers [29,35,36]. The phenomenon of plant growth inhibition as a direct consequence of water deprivation is intricately influenced by several pivotal factors, including but not limited to the inherent drought susceptibility characteristics of the plant species, the specific levels of water deficit in the environment, as well as the duration and intensity of the stress conditions over time [33,35,37,38]. The typical visible symptoms of leaf wilting caused by drought stress (Figure 5) were accompanied by a significant decrease in the leaf pigment content (Figure 1 and Figure S1). The reduction of leaf pigment content could be a consequence of accelerated pigment degradation [34,39] or decreased biosynthesis [40,41]. Reduced concentrations of chlorophylls and carotenoids along with a decrease in leaf water potential are typical consequences of drought that significantly alter plant physiological responses [42,43,44]. Under drought conditions, an impairment of leaf gas exchange processes is well documented [17,26,35,42]. Usually the reaction of wheat plants to water limitation is mainly attributed to a quick closure of the stomata, which in turn decreases the CO_2_ availability to the chloroplast and leads to a decline in An [45]. Accordingly, a noticeable decrease in stomatal conductance, net photosynthesis rate, and transpiration were detected in both cultivars (Figure 2 and Figure S1). However, we noticed a greater rate of net photosynthesis in cv. Gines as compared with cv. Fermer (Figure 2 and Figure S1) which could possibly be connected to its better drought tolerance. Sensitive-to-drought cultivars were reported to exhibit higher basal levels of photosynthesis and transpiration than those that are tolerant, but drought caused greater reduction in An and E in the susceptible variety [26,46]. In general, under conditions of severe drought, along with stomatal limitation, other constraints to lower photosynthesis appear to be non-stomatal restriction factors such as decreased efficiency of PSII and reduced activities of CO_2_-assimilating enzymes [45]. 

Drought resulted in a smaller reduction in carotenoids content as compared with chlorophyll in the drought-stressed plants of both cv. Gines and cv. Fermer (Figure 1). Carotenoids, vital pigments that serve as antioxidants, photo-protectants, and safeguard the plant membranes from oxidative damage, are also affected by drought and other stress agents [22,47]. Additionally, it has been demonstrated that chlorophyll *b* has a more remarkable decrease than chlorophyll *a* [48], which was also confirmed in our study. 

Photosynthetic pigments located in the antenna complexes of the thylakoid membranes absorb the energy of photosynthetically active radiation [49]. This energy is subsequently transferred as excitation energy to the reaction centers of PSII and PSI, where it is utilized for photochemical reactions. The donor part of PSII, in particular the Q_B_^−^ site on the D1 protein in the reaction center of PSII, is a crucial location for the disruption of the photosynthetic electron transport [50]. That part of photochemical energy which is not used for photochemistry is dissipated as heat and fluorescence of chlorophyll *a.* Drought stress resulted in alterations of the parameters of chlorophyll *a* fluorescence (Figure 3, Figure 4 and Figure S2). Maximal F_m_ and variable F_v_ fluorescence were found to decrease in both wheat cultivars, which indicates impairment in the electron transport chains that is reflected in decreased F_v_/F_m_ and F_v_/F_0_. The decline in the F_v_/F_0_ ratio probably indicates alterations in the structure of the thylakoid membranes [51,52] and signifies the affected donor side of PSII, as proposed earlier by other authors [50,53,54]. The decline in the F_v_/F_m_ ratio indicates a reduction in the maximal quantum yield of PSII, i.e., a decrease in the photochemical efficiency of the photosynthetic apparatus which correlates well with the decreased photosynthesis rate in plants grown under drought (Figure 2 and Figure 4). In addition, the F_0_/F_m_ ratio was increased, especially in the less drought-tolerant cv. Fermer, which implies intensified energy dissipation as heat that prevents overexcitation of the chlorophyll molecules, and protects the photosynthetic apparatus against photodamage under drought stress conditions [55].

After transferring the plants to a normal irrigation regime, the content of leaf pigment, net photosynthesis rate, transpiration, and stomatal conductance tended to recover. This tendency was better manifested in cv. Gines than cv. Fermer, which could be attributed to its higher tolerance to water deficit. It was reported that species with higher drought tolerance resumed their growth and photosynthetic activity faster than the drought-susceptible varieties [46,56]. Most of the parameters of chlorophyll *a* fluorescence in drought-stressed plants showed a tendency to recover to physiological levels, except that of F_0_, which was still increased, and this is reflected in the higher F_0_/F_m_ ratio (Figure 3, Figure 4 and Figure S2). Our results are in accordance with previously documented studies [57,58], where increased F_0_ is interpreted as an indicator of irreversible damage of PSII. Probably, this could be an outcome of drought-induced injuries to the PSII reaction center resulting in a decline in absorbed energy, an impairment in the basal quantum production of non-photochemical processes, and in successive increases in unutilized emitted light [59], which in our experiment persisted even during the recovery period. 

When analyzing the changes of parameters studied in plants treated with melatonin, it is obvious that with some exceptions such as the leaf pigment content its application did not significantly alter their levels under normal irrigation. However, in plants subjected to drought stress, the pre-application of melatonin (Mel-1 → Dr) retained chlorophyll pigments (Figure 1 and Figure S1) and increased the photosynthesis rate (Figure 2 and Figure S1), which is consistent with earlier information that melatonin treatment was capable of preserving higher concentrations of leaf pigments in stressed plants [21,60,61]. Our findings demonstrated that, in comparison to the drought-treated plants, the administration of melatonin mitigated the detrimental effects of drought stress and improved the content of chlorophylls and carotenoids (Figure 1 and Figure S1). These findings show that melatonin treatment (Mel-1 → Dr) possibly improved either the biosynthesis of pigments or decreased their degradation, which was caused by drought stress. Higher chlorophyll content contributes further to an improved photosynthetic rate, although no significant effect was noticed on transpiration rate and stomatal conductance (Figure 2 and Figure S1). The lower reduction of net photosynthesis in melatonin-treated plants suggested that they can be more adaptive to drought stress and could be more capable of maintaining their physiological functions. In addition, melatonin pre-treated plants (Mel-1 → Dr) showed better plant fitness under drought stress as compared with nontreated plants (Figure 5), which confirms the beneficial effect of melatonin application. As well as the favorable effects of exogenous melatonin on improving the photosynthesis rate by lowering the rate of chlorophyll degradation, increasing stomatal conductance, regulating stomata aperture, and intensifying CO_2_ assimilation [60,62,63], it can also enhance PSII efficiency [55]. The research of Ye et al. [64] confirmed that pre-treatment with melatonin supported the improvement in the photosynthetic and transpiration rates, stomatal conductance, parameters of chlorophyll *a* fluorescence, and leaf pigment content in maize plants subjected to drought. In addition, in tomato plants (*Lycopersicon esculentum* Mill.) melatonin enhanced the quantum yield both by alleviating photo-oxidative damage and by assisting the reparation of photo-oxidatively damaged D1 protein [65]. Likewise, melatonin-pretreated seedlings of rice (*O. sativa* L.) showed enhanced efficiency of PSII [66]. In accordance with these findings, we also found that pre-treatment with melatonin (Mel-1 → Dr) maintained better PSII photosynthetic efficiency, which is represented by higher levels of F_v_/F_m_ and F_v_/F_0_ ratios, along with lower values of F_0_/F_m_ as compared with drought-treated-only plants (Figure 3, Figure 4 and Figure S2). Our data demonstrated that melatonin pre-treatment (Mel-1 → Dr) was able to defend the photosynthesis machinery from damage due to water deficit. After restoring normal irrigation, melatonin pre-application (Mel-1 → Dr) accelerated the recovery of the leaf pigment content, stomatal conductance, photosynthetic and transpiration rate, and PSII photosynthetic performance, which suggests that it could also foster the reparation of drought-induced injuries of the photosynthetic system [17,64,67]. After re-hydration, melatonin-treated seedlings (Mel-1 → Dr) recovered more rapidly than the untreated plants (Figure 5). 

Similar to the pre-treatment with melatonin (Mel-1 → Dr), we found a drought-stress-attenuating effect on photosynthetic performance due to post-drought melatonin treatment (Dr → Mel-2), but this effect was weaker than that of Mel-1 → Dr. However, even when applied at a later phase it was capable of mitigating the negative consequences of drought and improving growth and photosynthesis-related parameters in post-drought treated wheat as compared with plants subjected to drought only (Figure 1, Figure 2, Figure 3 and Figure 4). Since the plants that were administered with melatonin exhibited higher values of chlorophyll after rehydration as compared with drought-only treated plants (Figure 1), we can hypothesize that melatonin post-drought application (Dr → Mel-2) could possibly be involved in de novo biosynthesis of the photosynthetic pigments. Evidence that melatonin can improve chlorophyll biosynthesis was reported earlier in pea plants [47]. Additionally, melatonin was reported to upregulate several genes related to chlorophyll biosynthesis in tomato, celery, and wheat plants [25,68,69], and to maintain high levels of chlorophyll precursors in mallow [24]. Further, it could be speculated that post-drought melatonin treatment (Dr → Mel-2) is probably involved in the overall repair processes of the photosynthetic apparatus and, as a result, it can support plant growth. Further in-depth investigations should be undertaken to prove these assumptions.

In conclusion, our findings showed that drought provoked significant deviations from the normal physiological state of the studied parameters in both cultivars, which were more pronounced in cv. Fermer. After restoring irrigation, cv. Gines regained the normal level of most of the studied parameters, while in cv. Fermer they remained below the control. This could be attributed to its lower drought tolerance. Under drought conditions (5D Dr) melatonin pretreatment increased the content of chlorophyll and carotenoids, maintained better chlorophyll *a* fluorescence characteristics, and improved net photosynthetic rate in both cultivars, which suggests a protective effect against drought-induced damage to photosystems. Additionally, after resuming irrigation, melatonin application prior to drought facilitated the recovery of photosynthesis-related parameters in both cultivars. When melatonin was applied after drought, although the effect was weaker, it was also capable of accelerating the recovery of photosynthesis-related parameters and improving the performance of the drought-treated plants.

## 4. Materials and Methods

### 4.1. Plant Material, Growth Conditions, and Treatments

**Scheme 1:**
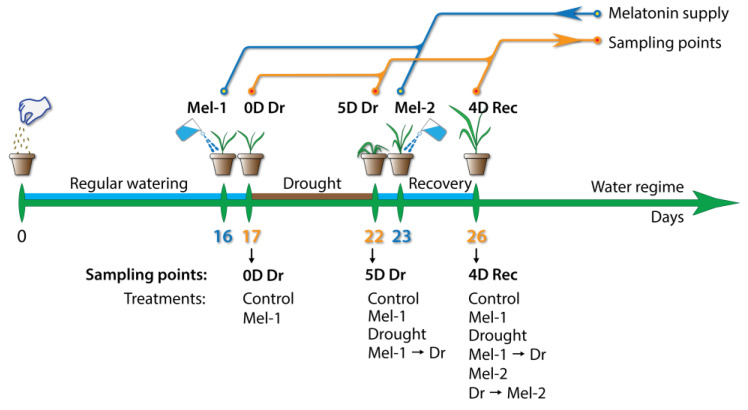
Timeline of the experiment: plant growth, treatments, sample collection, and measurement points.

The cv. Gines and cv. Fermer varieties of wheat (*Triticum aestivum* L.) used in this study are Bulgarian cultivars created at the Institute of Plant Genetic Resources, “K. Malkov”, Sadovo (Bulgaria). Cv. Gines was formerly characterized as more tolerant to drought than cv. Fermer [28,29]. The plants were cultivated in pots containing 560 g of 1:3 mixture of quartz sand:leached meadow cinnamon soil (pH 6.2). Each pot contained 20 seedlings and each treatment set consisted of six containers. The growth chamber parameters were as follows: temperature of 22 °C during the day and 19 °C at night; 60% relative air humidity; photoperiod of a 14 h day and 10 h night; artificial illumination supplying 200 μmol m**^−^**^2^ s^−1^ photon flux density. The level of soil moisture was kept at 75% of the field soil capacity by daily weighting the pots and regular watering. Young (17-day old) seedlings with a fully developed second leaf were subjected to drought stress for five days by withholding watering. Twenty-four hours before the beginning of the stress program, some of the plants were root supplemented with 10 mL water solution of 75 µM melatonin (Mel-1). The same treatment was administered to another set of plants, but 24 h after the end of the drought period (Mel-2). Following the end of the drought period, the normal irrigation regime was restored, and the plants were allowed to recover for four days. The control plants (watered with H_2_O or melatonin only) were regularly irrigated throughout the entire experiment (Scheme 1). 

To analyze the content of the photosynthetic pigments, samples from above-ground sections were collected at the beginning and at the end of drought stress, as well as after four days of the recovery period. At the same experimental points, photosynthesis and transpiration rate, stomatal conductance, and chlorophyll *a* fluorescence parameters were measured in vivo.

### 4.2. Leaf Pigment Content

Measurements of chlorophyll and carotenoids were conducted following the method outlined by Arnon [70]. Leaf pigments were extracted from about 30 mg of fresh leaf material in 5 mL of 80% acetone. The homogenates were spun at 5000× *g* for 5 min using a Sigma 2–16 K refrigerated centrifuge (SciQuip, Wem, UK). The supernatants obtained were diluted to 5 mL with 80% acetone to compensate for any evaporation that may have occurred. A Multiskan Spectrum spectrophotometer (Thermo Electron Corporation, Vantaa, Finland) was used to measure the absorbance at 460, 645, and 663 nm. The results were calculated on the basis of the dry weight of material collected from the same treatment groups, which was dried for 72 h in an oven at 104 °C. 

### 4.3. Parameters of Leaf Gas Exchange

Mature second leaves from different plants were used for conducting measurements of the leaf gas exchange rate. Before taking measurements, the leaf surface was left undisturbed to prevent stomata closure, as the leaves had already been acclimated to the environment. The changes in An, E, and Gs were monitored with a portable infrared gas analyzer system Li6400 from LI-COR Biosciences Inc. (Lincoln, NE, USA) fitted with an artificial light chamber (LI6400-02) and a 10L buffer to stabilize CO_2_ and H_2_O levels in the incoming air flow. The system was calibrated before measurement, following the manufacturer’s recommended standard procedure [71]. Measurements were conducted from 10:00 h to 14:00 h under the following controlled settings: actinic PAR of 200 μmol m^−2^ s^−1^ photon flux density, relative air humidity ranging from 40 to 45%, air temperature 25 °C, and air flow rate of 200 µmol s^−1^. 

### 4.4. Parameters of Chlorophyll a Fluorescence

In vivo measurements of chlorophyll *a* fluorescence were conducted with a Hansatech Plant Efficiency Analyzer—PEA MK2 (Hansatech Instruments Ltd., Kings Lynn, UK). The device includes a group of red LEDs that emit light at a peak wavelength of 650 nm. The plants used in the experiment were left to adapt to darkness for 30 min before measurement. Chlorophyll *a* fluorescence was measured in 10 mature leaves, which were exposed to red actinic light with a photon flux density of 3500 µmol m^−2^ s^−1^. The parameters measured included minimal fluorescence (F_0_) at time t = 0 when all PSII reaction centers are open, maximal fluorescence (F_m_) when all PSII reaction centers are closed, and maximal variable fluorescence (F_v_), which were then used for analyzing the efficiency of primary photochemical reactions (F_v_/F_0_—electron transport outside Q_A_^−^), the maximal quantum yield of PSII (F_v_/F_m_), and the quantum efficiency of heat dissipation (F_0_/F_m_).

### 4.5. Statistical Analysis

The data come from two separate trials carried out with a minimum of six replicas each. The figures show average values with standard error (±SE). A one-way ANOVA with Duncan’s post-hoc multiple range test was used to assess the significance of variations between the treatments in each sampling point at a level of *p* ≤ 0.05.

## Data Availability

Data available on request.

## Supplementary Materials

The following supporting information can be downloaded at: https://www.mdpi.com/article/10.3390/plants13233414/s1. Figure S1. Spider plot of chlorophyll *a* (Chl a), chlorophyll *b* (Chl b), carotenoids (Car), net photosynthesis rate (An), transpiration rate (E), and stomatal conductance (Gs) at the fifth day of drought (A, B) and at the fourth day of recovery (C, D) in wheat cv. Fermer (A, C) and cv. Gines (B, D). The values of the parameters are normalized to the control. Figure S2. Spider plot of minimal fluorescence (F_0_), maximal fluorescence (F_m_), variable fluorescence (F_v_), maximal quantum yield of photosynthesis (F_v_/F_m_), electron transport outside Q_A_^−^ (F_v_/F_0_) and quantum efficiency of energy dissipation(F_0_/F_m_) at the fifth day of drought (A, B) and at the fourth day of recovery (C, D) in wheat cv. Fermer (A, C) and cv. Gines (B, D). The values of the parameters are normalized to the control.
