# Supplementary material for: Changes of Photosynthetic Parameters in Melatonin-Treated Wheat Subjected to Drought
Source: Plants (Basel). 2024 Dec 5;13(23):3414. doi: 10.3390/plants13233414 (PMC11644088; doi:10.3390/plants13233414)
Supplement: Supplementary file 1 [file plants-13-03414-s001.zip › plants-3317587-supplementary.pdf]

Supplementary Figures to article:

Changes of Photosynthetic Parameters in Melatonin-Treated Wheat Subjected to Drought

Dessislava Todorova [1], Svetoslav Anev [2] , Martin Iliev [1], Margarita Petrakova [1] and Iskren Sergiev [1,*]

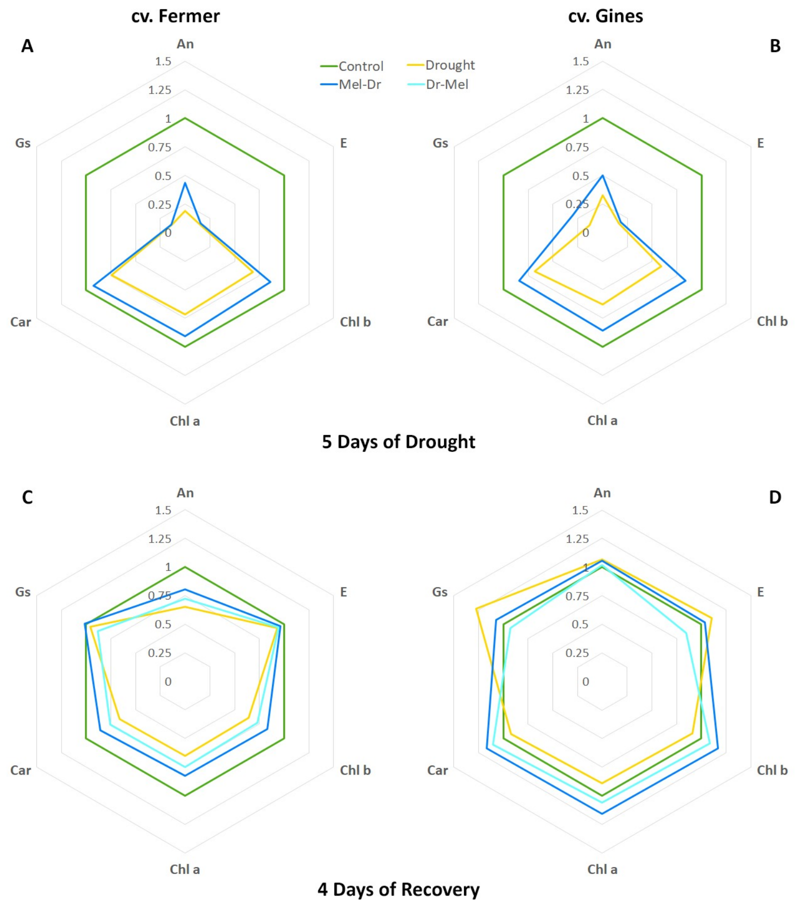

Figure S1. Spider plot of chlorophyll *a* (Chl a), chlorophyll *b* (Chl b), carotenoids (Car), net photosynthesis rate (An), transpiration rate (E), and stomatal conductance (Gs) at the fifth day of drought (**A**, **B**) and at the fourth day of recovery (**C**, **D**) in wheat cv. Fermer (**A**, **C**) and cv. Gines (**B**, **D**). The values of the parameters are normalized to the control.

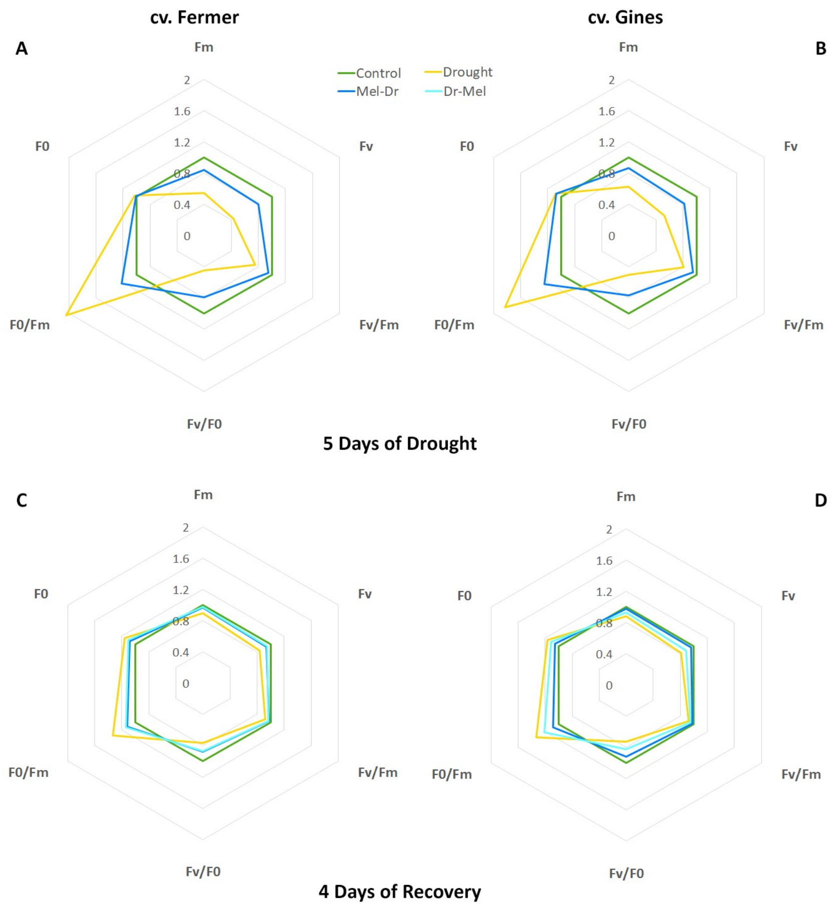

Figure S2. Spider plot of minimal fluorescence (F_0), maximal fluorescence (F_m), variable fluorescence (F_v), maximal quantum yield of photosynthesis (F_v/F_m), electron transport outside Q_A^- (F_v/F_0) and quantum efficiency of energy dissipation(F_0/F_m) at the fifth day of drought (**A**, **B**) and at the fourth day of recovery (**C**, **D**) in wheat cv. Fermer (**A**, **C**) and cv. Gines (**B**, **D**). The values of the parameters are normalized to the control.
